# Lower Extremity Nerve Decompression for Superimposed Peripheral Neuropathy in Diabetes: Awareness among Medical Professionals

**DOI:** 10.1097/PRS.0000000000012200

**Published:** 2025-05-13

**Authors:** Nadine Boers, Isabelle M. L. P. Kamm, Manuel Castro Cabezas, Wynand B. Melenhorst, Willem D. Rinkel, J. Henk Coert

**Affiliations:** Utrecht, Rotterdam, and Zeist, the Netherlands; From the 1 Department of Plastic, Reconstructive, and Hand Surgery, Utrecht University Medical Center, the Netherlands; 2 Department of Internal Medicine, Center for Diabetes, Endocrinology, and Vascular Medicine, Franciscus Gasthuis & Vlietland Hospital; 3 Department of Internal Medicine and Endocrinology, Erasmus University Medical Center; 4 Department of Plastic, Reconstructive, and Hand Surgery, Diakonessenhuis; 5 Julius Clinical.

## Abstract

**Background:**

In 2009, a Dutch survey disclosed that 23% of medical professionals involved in diabetes care acknowledged the role of superimposed nerve compression in diabetic sensorimotor polyneuropathy (DSP) symptoms, although less than 10% were aware of the potential value of nerve decompression surgery. The authors’ current aim was to assess whether awareness changed since this survey, together with an updated review of the literature.

**Methods:**

A cross-sectional, national, multiinstitutional survey-based study was conducted among professionals from different specialties in the Netherlands, including general practitioners, endocrinologists, neurologists, plastic surgeons, vascular surgeons, orthopedic surgeons, neurosurgeons, anesthesiologists, diabetes specialist nurses, and podiatrists.

**Results:**

Among the 730 respondents, 84% confirmed their involvement in diabetes care. Fifty-seven percent (versus 23% in 2009) stated being aware of the concept that superimposed nerve compressions play a role in the symptoms of DSP, and 74% (previously 60%) believed that this could be the case. In their communication to patients, 78% (versus 45%) of the professionals explained that DSP was irreversible. Thirty percent (versus 3%) reported to refer patients to a surgeon. The reviewed literature reports an encouraging effect regarding pain reduction and patient-reported outcome measures, albeit with inconsistencies in outcomes related to sensibility (static 1-point and 2-point discrimination) and nerve conduction parameters.

**Conclusions:**

In the past 13 years, studies reporting positive effects of surgery on both patient-reported outcomes and postoperative pain have raised an increased awareness on lower extremity neuropathy and lower extremity nerve decompression surgery, with significantly more referrals to surgeons. However, long-term outcomes and appropriate patient selection criteria for lower extremity nerve decompression surgery are still needed.

Diabetes mellitus (DM) is a highly prevalent metabolic disorder, affecting approximately 9.3% of the global population and is expected to rise even further in the coming years.^1^ With the increasing incidence of DM, an increase in diabetes-related complications is expected, resulting in more years lived in disability (ie, total health loss).^2^ One of the most critical and frequent complications of DM is diabetic sensorimotor polyneuropathy (DSP), a heterogeneous condition that manifests in various forms affecting both the central and peripheral nervous systems.^3^ DSP may lead to disabling neuropathic pain, sensory phenomena, and decreased sensibility, and it is the main driver to diabetic foot complications, such as ulceration and Charcot neuroarthropathy, frequently resulting in lower-limb amputation and mortality.^4^

Historically, DSP was considered to be an irreversible condition.^5,6^ Consequently, treatment strategies primarily focus on prevention by optimizing cardiovascular risk factors, glycemic control, and patient education.^7^ Despite these efforts, the global numbers of diabetic foot complications continue to rise.^8^ Nevertheless, studies have highlighted the reversibility of DSP-associated symptoms using peripheral nerve decompression, in light of the “double crush” hypothesis. Introduced by Adrian R. M. Upton, MD, and Alan J. McComas, MD, in 1973, this hypothesis suggests that localized compression at 1 nerve site can affect the nerve’s overall functionality, making it more susceptible to damage at another site.^9^ It was this hypothesis that stimulated A. Lee Dellon, MD, to further explore the relationship between diabetes and entrapment neuropathy in the early 1990s. As diabetes is the first “crush,” more entrapment neuropathies (second crush) are observed in diabetic subjects.^10^ Still, the scientific community’s view on lower extremity nerve decompression (LEND) surgery remains one of skepticism.^11^

In 2009, Melenhorst et al. reported that less than 10% of medical professionals primarily involved in diabetes care were aware of the potential benefit of LEND surgery in diabetes and also that only 3% were being referred for surgical management.^12^ The body of literature on the subject has expanded significantly since 2009.^13^ Therefore, our aim, based on repeated survey measurements, was to assess whether awareness on this subject has changed since 2009. We present this together with an overview of the current body of literature.

## PATIENTS AND METHODS

### Survey on Awareness among Health Care Providers

This cross-sectional, observational, descriptive, survey-based study was conducted among 1700 diabetes care providers, including a random selection of professionals from different specialties in the Netherlands. The questionnaire was conducted among general practitioners, endocrinologists, neurologists, plastic surgeons, vascular surgeons, orthopedic surgeons, neurosurgeons, anesthesiologists, diabetes specialist nurses, and podiatrists.

The questionnaire was anonymous and self-administered, including all questions from the 2009 version, with some additional questions for further clarification (Table 1). The questionnaire was sent by e-mail.

**Table 1.** Overview of Questionnaire Outcomes

Medical Specialist	No.	Involved in Diabetic Care (%)	Aware of Theory (%)^a^	Aware of Literature (%)^b^	Thinks Compression May Play a Role (%)^c^	Aware of Possible Value of Surgery (%)^d^	Explains DSP is Irreversible (%)^e^	Refers Patient to Nonsurgical Specialty (%)^f^	Refers Patient to Surgeon (%)^g^
General practitioners	121	98	32	2	74	7	88	63	18
Endocrinologists	44	96	73	14	75	43	71	65	19
Neurologists	77	97	60	8	56	31	94	33	57
Anesthesiologists	50	100	54	16	60	22	80	45	24
Diabetes specialist nurses	95	100	48	7	84	15	78	—	—
Podiatrists	126	99	68	16	82	18	86	—	—
Plastic surgeons	70	49	94	47	97	91	57	—	—
Vascular surgeons	44	100	61	16	54	30	80	—	—
Orthopaedic surgeons	64	31	39	8	70	23	55	—	—
Neurosurgeons	39	31	64	23	62	51	74	—	—
Overall	730	84	57	16	74	29	78	34	30

a “Are you aware of the theory that nerve compression injury plays a part in the pathophysiology of diabetic peripheral polyneuropathy?”

b “Are you aware of the latest literature on nerve compression in of diabetic peripheral neuropathy and suspicion of compression polyneuropathy?”

c “Do you think that nerve compression injury may play a role in the development of diabetic neuropathy?”

d “Are you aware of the potential value of nerve decompression surgery in the treatment of peripheral diabetic neuropathy?”

e “Do you explain to the patient that peripheral diabetic neuropathy is an irreversible condition?”

f “Do you refer to other medical professionals when considering diabetic neuropathy?”

g “If you answered ’yes’ to the previous question, please specify the specialty.”

### Statistical Analyses

Data were analyzed using IBM SPSS statistics 27.0 (IBM Corp., Armonk, NY). A chi-square test was used to compare the 2024 outcomes to outcomes from 2009. For these comparisons, only specialties and questions included in the questionnaire from 2009 were included. Values of *P* < 0.002, using the Bonferroni correction, were considered statistically significant.

### Literature Search

We identified all clinical studies reporting outcomes on LEND surgery up to 2024, including case series, cohort studies, and randomized controlled trials. We extracted the selection criteria used for LEND surgery, the number of studies evaluating objective and subjective outcomes of LEND surgery, and the number of studies reporting an improvement after surgery, either compared with the preoperative outcomes or to the preoperative difference in the control group. Because heterogeneity of the reported outcomes did not allow further quantitative analyses, this approach was used to summarize a best-evidence synthesis.

## RESULTS

### Questionnaire Outcomes

Of the 730 respondents (overall response rate, 43%), 84% confirmed their involvement in diabetes care, with the lowest percentages reported by orthopedic surgeons (31%), neurosurgeons (31%), and plastic surgeons (49%). Other specialties indicated a 96% to 100% involvement.

Table 1 presents survey outcomes for individual specialties. Table 2 shows comparisons between 2009 and 2023, conducted between 4 specialties that were also surveyed in 2009. Significantly more specialists reported being aware of the concept of LEND surgery (53% in 2023 versus 23% in 2009; *P* < 0.002). Significantly more communicated to patients that DSP is irreversible (83% versus 45%; *P* < 0.002), and significantly more refer patients to a surgeon (31% versus 3%; *P* < 0.002).

**Table 2.** Overview of Questionnaire Outcomes of 2023 versus 2009

Year	Medical Specialists	No.	Involved in Diabetic Care (%)	Aware of Theory (%)^a^	Thinks Compression May Play a Role (%)^b^	Aware of Possible Value of Surgery (%)^c^	Explains DSP Is Irreversible (%)^d^	Refers to Surgeon (%)^e,f^
2009	General practitioners	101	100	19	53	6	45	3
	Endocrinologists	28	100	32	79	11	46	0
	Neurologists	23	78	35	48	22	57	9
	Diabetes specialist nurses	20	100	15	90	5	35	—
	Overall	172	97	23	60	9	45	3
2023	General practitioners	121	98	32	74	7	88^g^	18^g^
	Endocrinologists	44	96	73	75	43^g^	7h^g^	19^g^
	Neurologists	77	97^g^	60^g^	56	31	94^g^	57^g^
	Diabetes specialist nurses	95	100	48^g^	84	15	78^g^	—
	Overall	337	98	53^g^	72	24	83^g^	31^g^

a “Are you aware of the theory that nerve compression injury plays a part in the pathophysiology of diabetic peripheral polyneuropathy?”

b “Do you think that nerve compression injury may play a role in the development of diabetic neuropathy?”

c “Are you aware of the potential value of nerve decompression surgery in the treatment of peripheral diabetic neuropathy?”

d “Do you explain to the patient that peripheral diabetic neuropathy is an irreversible condition?”

e “Do you refer to other medical professionals when considering diabetic neuropathy?”

f “If yes, to which specialty?”

g Significance level (*P* < 0.002).

## LITERATURE REVIEW

Up to 2024, 31 clinical studies have reported outcomes on LEND surgery in diabetic patients.^14–49^ A total number of 13 clinical studies were included in the 2009 summary by Melenhorst et al.^12^

Sixteen of 31 clinical studies (52%) are case series,^16,18,20,21,24,26,29–32,36–39,41,47^ 10 studies (32%) are prospective cohort studies,^15,17,19,22,23,27,28,33–35^ and 5 studies (16%) are randomised controlled clinical trials.^14,40,42,48,49^ In addition, 2 Markov analyses^50,51^ and 2 cost-effectiveness analyses^52,53^ were published.

### Patient Selection

All clinical studies included patients with neuropathic symptoms, of which 12 studies (39%) specifically included patients with painful diabetic neuropathy.^14,18–22,24,26,31,33,40,41^ Table 3 displays the selection criteria of the included studies.

**Table 3.** Overview of Selection Criteria Used for LEND Surgery

Year	First Author	Positive Tinel Sign	DSP Symptoms	Pain	Good Vascularity	Decreased 2PD	Abnormal Cutaneous Threshold	HbA1c Controlled	NCS	Ulcer and/or Amputation
Case series										
1995	Wieman	+	+	+	+	–	–	–	–	–
2000	Caffee	–	+	–	+	–	+	–	–	–
2001	Tambwekar	–	–	–	+	–	+	–	–	+
2003	Wood	+	+	–	+	+	–	–	–	–
2004	Aszmann	–	+	–	–	+	+	–	–	–
2004	Biddinger	–	+	–	+	+	–	–	–	–
2005	Valdivia	+	+	–	+		–	+	–	–
2006	Ducic	+	+	–	–	–	–	–	–	–
2006	Siemionow	+	+	+	–	–	–	–	–	–
2012	Knobloch	+	+	+	+	–	+	–	–	–
2013	Nickerson	+	+	+	+	–	–	–	–	–
2017	Anderson	+	+	+	+	–	+	–	–	–
2018	Liao	+	+	+	+	+	–	+	–	–
2018	Wang	+	+	+	+	+	–	–	–	–
2021	Uemura	+	+	–	–	–	+	–	+	–
2023	Meyer-Marcotty	+	+	+	+	–	–	–	+	–
Prospective cohort										
1992	Dellon^a^	+	+	–	–	–	–	–	–	–
2000	Aszmann	+	+	–	–	–	–	–	–	–
2005	Rader	+	+	+	–	–	–	–	–	–
2008	Karagoz	+	+	–	+	–	–	–	–	–
2012	Dellon^a^	+	+	–	+	–	–	+	–	–
2013	Zhang	+	+	–	+	+	–	–	–	–
2014	Liao	+	+	+	+	+	–	–	–	–
2016	Yang	+	+	+	+	+	–	+	+	–
2021	Agarwal	+	+	–	–	–	+	–	–	–
2022	Fakkel	+	+	–	+	–	–	–	–	–
RCTs										
2014	Van Maurik	+	+	–	+	–	–	–	–	–
2019	Best	–	+	+	+	–	–	+	–	–
2022	Mahmoud	+	+	+	–	–	–	–	+	–
2023	Ma	+	+	–	–	–	–	+	+	–
2024	Rozen	+	+	–	–	–	–	+	+	–

+, articles using this as an inclusion criterion; –, articles not reporting this as an inclusion criterion; NCS, nerve conduction studies; RCTs, randomized controlled trials.

a Two articles using the same patient cohort (Dellon AL, Muse VL, Nickerson DS, et al. Prevention of ulceration, amputation, and reduction of hospitalization: outcomes of a prospective multicenter trial of tibial neurolysis in patients with diabetic neuropathy. *J Reconstr Microsurg*. 2012; 28:241– 246; and Dellon AL, Muse VL, Scott ND, et al. A positive Tinel sign as predictor of pain relief or sensory recovery after decompression of chronic tibial nerve compression in patients with diabetic neuropathy. *J Reconstr Microsurg*. 2012;28:235– 240).

The Tinel sign was the most commonly used clinical test, serving as a mandatory selection criterion in 26 of 31 studies (84%).^15–29,31–35,39–49^ Diminished 2-point discrimination (2PD) and an abnormal cutaneous threshold (static 1-point discrimination [1PD]) were used to select patients in 8 (26%)^18–20,22,25,29,30,38^ and 7 (23%)^16,17,21,36–38,41^ studies, respectively. One study used both 2PD and 1PD.^38^ Six studies (19%) selected patients based on abnormal nerve conduction studies,^16,22,40,47–49^ typically indicated by a reduced amplitude of the sural sensory response.^54^

Because an adequate peripheral vascularity could potentially create a more favorable environment for the decompressed nerve and also lower the risk of surgical-site–related problems (eg, infections), the majority of studies (20 studies [65%]) excluded patients with peripheral vascular disease.^14,15,18–26,29,30,32,34–37,41–47^ Twelve studies (38%) included patients with (previous) ulcers.^15,19,24–26,30,34,36–38,40,49^

### Patient-Reported Outcomes Measures

Table 4 summarizes the studies and outcome measures up to 2024. All 23 studies evaluating pain as outcome document a decrease in visual analog scale scores 6 to 56 months after surgery,^14,16–23,26,29–33,35,36,40–42,47–49^ although 5 of these studies (22%) did not specify the significance of their findings.^16,17,30,32,41^ Six of 31 studies (19%) evaluated quality of life, with 4 studies reporting a significant improvement 12 to 24 months after surgery.^15,20,22,49^ Two studies reported no differences 12 and 18 months after surgery, respectively.^14,42^ One study (3%) examined anxiety and depression and found these scores to improve significantly compared with the control group 24 months after surgery.^20^ Five studies (16%) applied clinical scoring tools.^14–16,25^ Among these, 4 studies (80%) reported significant improvements in the Toronto Clinical Scoring System at 6 and 18 months,^25,48^ the modified Toronto Clinical Scoring System at 12 months,^16^ and the Michigan Neuropathy Screening Instrument score at 13 months’ follow-up, respectively.^15^ However, 1 randomized controlled trial found no difference in the total neuropathy score 12 months after surgery.^14^

**Table 4.** Outcome Measures Reported in Clinical Studies up to 2023 versus 2009

Outcomes Evaluated	No. of Clinical Studies Evaluating Surgical Outcomes in 2009	No. of Clinical Studies Evaluating Surgical Outcomes in 2023	No. of Clinical Studies Reporting Improved Outcomes in 2023 (%)
Visual analog score	8	23	23 (100)
Quality of life	0	6	4 (75)
Clinical scoring tools	0	5	4 (80)
Semmes-Weinstein or PSDD	9	15	14 (93)
2-PD	8	13	10 (77)
Recovery of ulcus or prevention of ulcus or amputation	6	13	12 (92)
Reduction of pain medication	2	2	2 (100)
Nerve conduction studies	1	9	6 (67)
Quantitative sensory test	0	1	1 (100)
Ultrasound outcomes	0	4	3 (75)
Stability	3	6	4 (67)
Total	13	31	

PSDD, pressure-specified sensory device.

### Objective Outcome Measurements

In terms of objectively assessed outcomes, 13 studies (42%) evaluated 2PD,^14,17,19,23,27–31,33,42,48,55^ with 10 of these studies (77%) noting improvement 3 to 30 months after surgery.^17,19,23,27–31,48,55^ Fifteen studies (48%) assessed 1PD,^16,17,23,27,30–33,36,37,39,41,42,47,55^ with 14 of these studies (93%) reporting improvement 6 to 30 months after surgery.^16,17,23,27,30–33,36,37,39,41,42,47,55^ Balance was evaluated in 6 studies (19%)^21,32,36,39,41,42^ and was improved in 4 of these (67%) 6 to 18 months after surgery.^21,32,39,41^

A minority of studies evaluated preoperative and postoperative nerve conduction parameters,^14,19–21,25,26,40,46,48^ ultrasound,^19,20,40,45^ and quantitative sensory testing, respectively.^25^ Six of 9 studies (67%) evaluating nerve conduction reported significantly improved parameters compared with preoperative results^21,25,48^ or a control group 6 to 24 months after surgery.^19,20,40,48^ Intraoperative electromyography was performed in 1 study, showing immediate improvement 1 minute after decompressing the common fibular nerve in 82.6% of cases.^21^ Three of 4 studies (75%) evaluating ultrasound outcomes found a decreased cross-sectional area of the nerves after surgery when compared with the control group 6 to 24 months after surgery.^19,20,40^ Only 1 study evaluated quantitative sensory testing, observing cold and warmth sensory functions to significantly improve after surgery, which were almost restored to normal levels.^25^

One long-term promise of LEND surgery has been that it is to be able to reduce the recurrence of ulcerations, as evaluated by 13 studies (42%).^17,19,25,26,30,33,34,36–38,40,41,56^ Twelve of these (92%) reported decreased ulceration rates or no recurrences in the operative legs 6 to 32 months after surgery,^17,19,24–26,30,33,34,36–38,41^ although only 3 of these studies made comparisons with a nonoperative leg^24,38^ or nonoperative patients.^19^ One trial noted no difference in the incidence of foot ulcers when compared with nonoperative patients after 6 months. The authors reported that an improvement in ischemic manifestations after surgery was seen, but did not further specify this.^40^

Regarding the reduction of diabetic foot complications, 4 studies used existing literature data to model the potential cost-effectiveness of LEND surgery.^50–53^ All 4 studies concluded that it would reduce diabetic foot ulceration and amputations in the long term (up to 10 years), leading to improved survival, quality of life, and cost savings for society.

## DISCUSSION

Nerve decompression surgery has been reported for years to have the potential to (partially) reverse symptoms and prevent complications of neuropathy, with a growing evidence base for LEND surgery in more recent years. The reviewed studies report an encouraging effect regarding patient-reported outcome measures and pain reduction, albeit with inconsistencies in outcomes related to improvement in sensibility (eg, 2-PD) and nerve conduction parameters. In line with this, our survey data have shown that the predominant view among clinicians has indeed changed since 2009, with significantly more clinicians being aware of the concept of superimposed nerve compression and acknowledging the potential beneficial role of LEND surgery in the treatment of DSP-related symptoms.

The recent body of literature has improved awareness and encouraged clinicians to refer more patients for surgical treatment. The surveyed Dutch referral rate to surgeons of 30% is much closer to the reported incidence of tibial nerve compression among patients with DSP, which is estimated at 45%.^57^ Of these patients, 20% are reported to have 1 of 10 possible compression sites and 80% had compression at multiple sites.^15^ Although the majority of patients with DSP do not develop superimposed compression neuropathy, these percentages offer real hope, as accurate diagnosis provides a viable treatment option for the ones with nerve entrapment. However, with the absence of a standard for identifying patients with lower extremity nerve compression, patients potentially benefiting from LEND surgery are still overlooked.

The discrepancy between awareness and referral rate may be attributed to a few causes. First, the lack of guidelines and appropriate patient selection criteria may play a role. The Tinel sign is the most often advocated and studied diagnostic maneuver, used in 93% of studies reporting methods for diagnosing tarsal tunnel syndrome (unpublished data from a large systematic review on diagnosing tarsal tunnel diagnosis from Boers et al.). The Tinel sign is theorized to indicate sensory axonal damage through remyelination and axonal regeneration, making it a clinically relevant diagnostic tool, as it becomes positive with prolonged but reversible compression and negative when damage becomes irreversible.^58^ Although the Tinel sign may not strongly correlate with motor nerve regeneration,^59^ this limitation does not affect its suitability for identifying candidates for LEND surgery, where restoring sensory function remains the primary objective.

Second, it remains unclear which patient characteristics contribute to a better outcome after decompression surgery. Liao et al. proposed that the presence of focal pain or pain induced by mechanical stimuli are both predictors of a successful surgical outcome, although only 39% of included studies used pain as a selection criterion or distinguished between focal or diffuse pain.^19,20^ With regard to severity of nerve compression, 4 studies propose that an early stage of nerve injury (eg, a 2PD <20 mm^18^) is associated with better pain relief after external neurolysis.^18,30,55,58^ Three studies found a positive Tinel sign, which is suggested to be associated with a relatively early stage of nerve injury and is the most commonly used selection criterion, to predict pain relief after LEND surgery.^30,55,58^ In contrast, Biddinger and Amend suggested that documented axonal loss (eg, widening 2PD, not further specified), and thus a more advanced stage of nerve compression, was a positive predictor for better surgical outcomes.^30^ Although this is not confirmed by other studies, a substantial part (38%) of the studies also included patients with past or present ulcers, indicative of more advanced stages or even end-stage neuropathy.^15,19,24–26,30,34,36–38,40,49,60^ The great variety in selection criteria used in previous cohort studies and trials, together with the varying symptoms, severity, complications, and comorbidities of DSP, pose great challenges in comparing available data.

Another challenge when evaluating surgical outcomes, is the relatively short follow-up period reported in the studies. This may overlook potential long-term benefits that could result from LEND surgery, as nerve regeneration is a slow process. This is especially important considering reinnervation of the foot. For example, proximal decompressed nerves (eg, common peroneal nerve, tibial nerve at soleal sling) may need 1 to 1.5 years to reach their somatosensory target in the foot. Recent long-term (ie, 10 years) modeled outcomes suggest that LEND surgery may be both cost saving and cost effective compared with nonsurgical management, offering significant potential socioeconomic advantages.^50–53^ This particularly includes the result of reinnervating the plantar foot (tibial nerve) and thereby preventing reulceration. However, well-designed long-term real-world studies are needed to confirm this. To date, 3 randomized controlled trial (RCT) protocols have been published.^61–63^ Liao and colleagues compare decompression of the tibial nerve, common, and deep peroneal nerve to a nonsurgical group receiving current best care and plan to score pain, sensory function, motor function, and neural signal conduction in 40 patients at 6 months after LEND surgery.^62^ Daeschler and colleagues unilaterally decompress the tibial nerve and common peroneal nerve and compare this to the nondecompressed contralateral lower extremity. They plan to evaluate pain reduction, sensory function, quality of life, and analgetic use in 74 patients 12 months after surgery.^63^ An RCT by Rinkel et al. extended the observational period to 4 years, aiming to contribute real-life data to the current quality-of-life analyses and cost-effectiveness analyses.^50–53^ In the DeCompression trial, up to 5 sites of nerve entrapment in the lower extremity are decompressed. The surgery is compared with a current best care group of diabetic subjects receiving no additional nerve surgery.

Awareness is highest among plastic surgeons, who primarily perform this type of surgery in the Netherlands. However, these professionals typically do not routinely manage diabetes. Improving patient selection and referral patterns are needed to improve collaboration between the involved specialties. To address this, a Delphi study with stakeholders should be carried out to identify drivers for referral to identify the arguments of nonreferring specialists, to know the objectives of surgeons and overall awareness and expectations of all parties involved. This strategy will promote implementation of the surgery on a broader level when definitive conclusions can be drawn from the current RCTs on the subject. Thereby, outcomes of decent quality studies should be presented and published in nonsurgical journals. Hereby, results possibly find their way to the practice advisories of every specialty involved in diabetes care and convince or refute the ideas on LEND surgery in communities of specialties that are currently hesitant to underscore the effects of surgery in selected diabetic subjects.

Although we were able to collect data from many respondents, this study does have some limitations. First, we were not able to question the responders of 2009 with the same questionnaire. Therefore, our study could not identify learning effects on the topic by a direct comparison. However, because our study included more responders (730 versus 172), we are confident that these data can be used so that we can draw conclusions on the increased awareness on the subject. Second, we were not able to perform a nonresponders analysis on characteristics of people who did not complete our questionnaire. The response rate was determined by the patient following the survey link and finishing or not finishing the survey. Details on sex, hospital type, and specialism of nonresponders are lacking. Nonetheless, we believe that by including a large, random selection of both academic, teaching, and general hospitals in the Netherlands, our sample is representative of the survey population. Together with the internationally available data from previous study, we believe our data are generalizable to countries with comparable health systems. Still, we must consider the potential overestimation of the awareness, as we expect clinicians with a more profound interest in the subject are more likely to have completed the survey. Third, although this review provides an overview of the available data on LEND surgery in the context of diabetes-induced neuropathy, the inconsistent reporting of outcome measures, such as absolute numbers and statistical significance, increases the possibility of reporting bias. The abundance of mostly retrospective cohort studies in the literature may also introduce a publication bias, potentially leading to an overestimated effectiveness of LEND surgery for DSP. These issues underscore the need for well-designed prospective studies, which are preceded by publication of the study protocol in the literature.

## CONCLUSIONS

In the past 13 years, there has been a growing evidence base for LEND surgery with promising outcomes on patient-reported outcome measures and postoperative pain, but inconsistencies in outcomes related to improvement in sensibility, 2PD, and nerve conduction parameters still exist. In line with this, awareness of LEND surgery has increased significantly, and substantially more referrals to surgeons were reported, with referral rates being much more in line with the expected incidence of nerve compression in patients with DSP. However, more prospective studies are needed to evaluate long-term outcomes and improve patient selection criteria for LEND surgery.

## DISCLOSURE

The authors have no conflicts of interest to disclose. No funding was received for this work.
